# Temperature, mosquito feeding status and mosquito density influence the measured bio-efficacy of insecticide-treated nets in cone assays

**DOI:** 10.1186/s13071-024-06210-y

**Published:** 2024-03-28

**Authors:** Jilly Jackson Mseti, Masudi Suleiman Maasayi, Aidi Galus Lugenge, Ahmadi B. Mpelepele, Ummi Abdul Kibondo, Frank Chelestino Tenywa, Olukayode G. Odufuwa, Mgeni Mohamed Tambwe, Sarah Jane Moore

**Affiliations:** 1 Vector Control Product Testing Unit, Environmental Health and Ecological Science Department, Ifakara Health Institute, 74, Bagamoyo, Tanzania; 2 School of Life Sciences and Bioengineering, The Nelson Mandela African Institution of Science and Technology (NM-AIST), 447, Arusha, Tanzania; 3 Vector Biology Unit, Epidemiology and Public Health Department, Swiss Tropical and Public Health Institute, Kreuzstrasse 2, Allschwil, 4123 Basel, Switzerland; 4 University of Basel, Petersplatz 1, 4001 Basel, Switzerland; 5 MRC International Statistics and Epidemiology Group, London School of Hygiene and Tropical Medicine (LSHTM), London, WC1E 7HT UK

**Keywords:** Cone bioassay, ITNs, PBO, Bioefficacy, *Anopheles funestus*, Temperature, Feeding status, Mosquito density, Vector control, Tanzania

## Abstract

**Background:**

The WHO cone bioassay is routinely used to evaluate the bioefficacy of insecticide-treated nets (ITNs) for product pre-qualification and confirmation of continued ITN performance during operational monitoring. Despite its standardized nature, variability is often observed between tests. We investigated the influence of temperature in the testing environment, mosquito feeding status and mosquito density on cone bioassay results.

**Methods:**

Cone bioassays were conducted on MAGNet (alphacypermethrin) and Veeralin (alphacypermethrin and piperonyl butoxide (PBO)) ITNs, using laboratory-reared pyrethroid-resistant *Anopheles funestus *sensu stricto (FUMOZ strain) mosquitoes. Three experiments were conducted using standard cone bioassays following WHO-recommended test parameters, with one variable changed in each bioassay: (i) environmental temperature during exposure: 22–23 °C, 26–27 °C, 29–30 °C and 32–33 °C; (ii) feeding regimen before exposure: sugar starved for 6 h, blood-fed or sugar-fed; and (iii) mosquito density per cone: 5, 10, 15 and 20 mosquitoes. For each test, 15 net samples per treatment arm were tested with four cones per sample (*N* = 60). Mortality after 24, 48 and 72 h post-exposure to ITNs was recorded.

**Results:**

There was a notable influence of temperature, feeding status and mosquito density on *An. funestus* mortality for both types of ITNs. Mortality at 24 h post-exposure was significantly higher at 32–33 °C than at 26–27 °C for both the MAGNet [19.33% vs 7%; odds ratio (OR): 3.96, 95% confidence interval (CI): 1.99–7.87, *P* < 0.001] and Veeralin (91% vs 47.33%; OR: 22.20, 95% CI: 11.45–43.05, *P* < 0.001) ITNs. Mosquito feeding status influenced the observed mortality. Relative to sugar-fed mosquitoes, The MAGNet ITNs induced higher mortality among blood-fed mosquitoes (7% vs 3%; OR: 2.23, 95% CI: 0.94–5.27, *P* = 0.068) and significantly higher mortality among starved mosquitoes (8% vs 3%, OR: 2.88, 95% CI: 1.25–6.63, *P* = 0.013); in comparison, the Veeralin ITNs showed significantly lower mortality among blood-fed mosquitoes (43% vs 57%; OR: 0.56, 95% CI: 0.38–0.81, *P* = 0.002) and no difference for starved mosquitoes (58% vs 57%; OR: 1.05, 95% CI: 0.72–1.51, *P* = 0.816). Mortality significantly increased with increasing mosquito density for both the MAGNet (e.g. 5 vs 10 mosquitoes: 7% vs 12%; OR: 1.81, 95% CI: 1.03–3.20, *P *= 0.040) and Veeralin (e.g. 5 vs 10 mosquitoes: 58% vs 71%; OR 2.06, 95% CI: 1.24–3.42, *P* = 0.005) ITNs.

**Conclusions:**

The results of this study highlight that the testing parameters temperature, feeding status and mosquito density significantly influence the mortality measured in cone bioassays. Careful adherence to testing parameters outlined in WHO ITN testing guidelines will likely improve the repeatability of studies within and between product testing facilities.

**Graphical Abstract:**

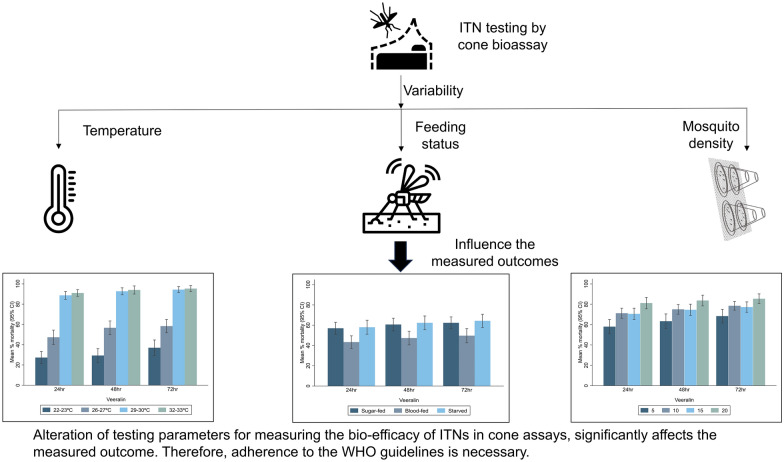

**Supplementary Information:**

The online version contains supplementary material available at 10.1186/s13071-024-06210-y.

## Background

In the ongoing battle against malaria, insecticide-treated nets (ITNs) are an elegant and effective tool. They provide a physical barrier against mosquito bites and deliver insecticide to mosquitoes attracted to the human hosts sleeping beneath them that ultimately leads to a reduction in mosquito vector population size and age [1, 2]. ITNs have significantly contributed to the global effort to reduce malaria-related morbidity and mortality [3, 4]. Their widespread distribution and use are integral components of malaria control programs in endemic regions [4, 5]. Prior to the distribution of new ITNs for public health use, a comprehensive assessment of their safety and efficacy is essential, following the standards and criteria established by the WHO [6, 7]. The WHO has issued specific guidelines for the laboratory tests and field trials required to generate the data needed to secure WHO Prequalification (PQ) listing [8], as well as for evaluating the durability of ITNs, which is crucial for verifying their sustained effectiveness after extended use [9, 10].

The cone test is a standardized bioassay and a useful tool for characterizing the biological availability and potency of active ingredients on the surface of an ITN under controlled laboratory conditions by holding mosquitoes in forced contact with the ITN to measure bioefficacy (knockdown and mortality) [11]. The test is commonly used to evaluate ITNs as part of a prequalification data dossier as well as part of post-market surveillance [12]. The standard procedure for cone bioassay involves exposing five non-blood-fed female mosquitoes aged 2–5 days per cone to an ITN sample that is pinned onto a bioassay board (25 × 25 cm) for testing. Following a 3-min exposure to the ITN sample at 27 °C ± 2 °C and 75 ± 10% relative humidity, the mosquitoes are transferred into holding cups with access to a 10% sugar solution. The test generates two outputs: (i) knockdown, which is the proportion of incapacitated mosquitoes after contact with a sub-lethal dose of insecticides that results in the inability of the mosquito to fly or maintain normal posture measured 60 min after exposure; and (ii) 24 h mortality, which is the proportion of dead mosquitoes measured at 24 h post-exposure. Both knockdown and 24 h mortality are the immediate toxicity indicators of neuro-toxic insecticides [11, 13]. Longer holding times are sometimes used for slower acting ITN active ingredients (AI).

While the cone bioassay is designed to accurately measure ITN bioefficacy, variations in results are often observed between the tests [14]. Therefore, there is a critical need to comprehensively investigate the potential impact of variations in the parameters involved in testing. Included in the key parameters that have shown to influence the outcome of insecticide exposure are temperature [15], mosquito feeding status [16] and mosquito density (Seth Irish, personal communication). Temperature has a fundamental effect on both the behavior and physiology of mosquitoes, playing a critical role in modulating insecticide metabolism and resulting toxicity [17–19] and affecting their interactions with vector control tools [17, 20]. The feeding status of mosquitoes represents another significant experimental factor that can significantly affect their interaction with insecticides [21] as it provides them with additional resources if they have been under nutritional stress [22] and changes their metabolism [23]. Blood-feeding makes the insects heavy, which may impact their ability to avoid an irritant active ingredient, and diuresis causes a downregulation of transcripts associated with oxidative metabolism [24]. However, the precise effects of these three parameters within the context of the cone bioassay have not been comprehensively explored. Therefore, this study was conducted to assess how alterations in temperature, mosquito feeding status and mosquito density, respectively, may impact the outcome of cone bioassays conducted against pyrethroid ITNs and ITNs impregnated with the synergist piperonyl butoxide (PBO) that is currently used on ITNs to restore the efficacy of pyrethroids in metabolically resistant mosquitoes through blocking cytochrome P450 detoxification enzymes [25, 26]. *Anopheles funestus* sensu stricto (*An. funestus* s.s.) mosquitoes were used for testing. This strain is resistant to pyrethroids through upregulation of metabolic enzymes [16] and is among the major transmitters of malaria in Tanzania [27, 28].

## Methods

### Study area

The experiments were conducted at the facility of the Vector Control Product Testing Unit (VCPTU) of the Ifakara Health Institute (IHI) located in Bagamoyo at (6.446°S, 38.901°E), Tanzania from June to July 2023.

### Mosquitoes

Laboratory-reared pyrethroid-resistant *An. funestus* (Fumoz strain) mosquites were used in the experiments. This mosquito strain is metabolically resistant to pyrethroids [16]. The strain was confirmed to show 50% mortality to alpha-cypermethrin at a 1 × discriminating concentration (0.05%), but its susceptibility was 100% restored through pre-exposure to PBO (4%) at the time of experimentation. The mosquitoes were nulliparous females aged between 3 and 5 days. Mosquitoes are maintained in the insectary at 27 ± 2 °C and 40–100% relative humidity (RH), under ambient conditions (12:12 light:dark) following MR4 Guidelines [29]. For colony maintenance, larvae are fed Tetramin fish flakes, and adult mosquitoes are provided with a 10% sugar solution and cow blood through membrane feeding to facilitate egg laying.

### Test products

Two classes of unused and unwashed ITNs (MAGNet and Veeralin; V.K.A. Polymers Pvt. Ltd, Karur, Tamil Nadu, India) were tested using the cone bioassays. The MAGNet ITN is made from 150-denier monofilament yarn consisting of high-density polyethylene impregnated with alpha-cypermethrin at a concentration of 5.8 g/kg (261 mg/m^2^). The Veeralin ITN is composed of 130-denier fabric and is impregnated with alpha-cypermethrin at a concentration of 6.0 g/kg (216 mg/m^2^) and PBO at 2.2 g/kg (79.2 mg/m^2^). Safi Net, an ITN made of polyester (A to Z Textile Mills, Tanzania) was used as a negative control to evaluate the quality of the bioassays.

### Net subsamples preparation

Five net sample pieces (25 × 25 cm) were cut from three panels of each net, resulting in 15 pieces per net. Each net piece was uniquely labeled, wrapped in aluminum foil and then stored at 4 °C in a temperature-controlled refrigerator.

### Study procedures

#### Cone tests

Cone tests were conducted between 1:00 p.m. and 4:00 p.m. each day, outside of the time when the circadian rhythm might affect the metabolism of pyrethroids [30]. For the temperature experiment, cone tests were conducted between 1:00 p.m. and 8:00 p.m. because the experiments were conducted over a wide range of temperatures, ranging from low temperatures to high temperatures, in a single day and it took time to change the room temperature. ITN samples were removed from the fridge and kept in the incubator for 1 h, then laid over the testing board 1 h before the experiment started so that the samples could return to room temperature. The board has four holes with a diameter of 12 cm that are regularly spaced, which allows for four cone replicates to be conducted per sample of material. Standard test cones made of translucent polyvinyl chloride (PVC) 12 cm in diameter were used with a plastic bung to discourage mosquitoes from resting on it. The purpose of the holes in the frame is to ensure that test mosquitoes are in contact with the test material and not the frame. Mosquitoes were exposed for 3 min in all tests. After exposure, the mosquitoes were aspirated into a paper cup, with a separate cup for each replicate, and then kept at 27 ºC ± 2 ºC and 60–100% RH and provided with 10% sucrose solution. For all tests, 24-, 48- and 72-h mortality were recorded.

### Effect of exposure temperature on *An. funestus* mortality

Cone bioassays were performed for 3 consecutive days under four different temperature conditions: 22–23 °C, 26–27 °C, 29–30 °C and 32–33 °C. The desired temperatures were maintained using a 11-Fin oil heater (Tronic) and a DUAL Inverter air conditioner (AC) (LG Electronics), and relative humidity was maintained at 75% ± 20% using a model B 25 E Design Humidifier (Trotec GmbH, Heinsberg, Germany) and four bowls measuring (diameter 30 cm) filled with water placed on the floor of the testing facility. A Tinytag [Gemini Data Loggers (UK) Ltd, Chichester, UK] was placed close to the testing board to monitor the environmental conditions throughout the tests.

For each test, five sugar-fed mosquitoes were exposed for 3 min in each cone, with four cones per net piece. Sixty cone bioassays were conducted per net type for each temperature range. For ease of working, mosquitoes were exposed in the order of increasing temperature, starting at the lowest temperature and progressing to the highest temperature each day. Mosquitoes were kept at 27 ºC ± 2 ºC and 60–100% RH and provided with 10% sucrose solution before and after testing.

### Effect of feeding status on *An. funestus* mortality

Mosquitoes at various feeding statuses were tested in experiments conducted on 3 consecutive days: (i) starved (starved for 6 h before exposure); (ii) blood-fed (fully fed on human arm 24 h before exposure); and (iii) sugar-fed (maintained on sugar until exposure). For each test, five mosquitoes from each feeding group were exposed for 3 min to each cone, with four cones per net piece. Temperature and humidity were maintained at 27 ºC ± 2 ºC and 75% ± 10%, respectively, in all the experiments. Sixty cone bioassays were conducted per net type for each feeding status.

### Effect of mosquito density on *An. funestus* mortality

Cone bioassays were performed for 3 consecutive days using 5, 10, 15 and 20 sugar-fed mosquitoes exposed to each cone in the WHO cone bioassay. For each test, mosquitoes from each density group were exposed for 3 min to each cone, with four cones per net piece. Temperature and humidity were maintained at 27 ºC ± 2 ºC and 75% ± 10%, respectively, in all the experiments. Sixty cone bioassays were conducted per net type for each density.

### Data management and statistical analysis

Data were collected into hard copy and then double entered into Microsoft Excel before being imported into STATA 17 for analysis. Descriptive statistics were conducted to estimate the mean percentage and 95% confidence intervals (CI) of mosquito mortality at 24, 48 and 72 h for each experiment.

Mixed effect logistic regression for grouped data with a binomial distribution and logit function was used to assess the effect of temperature, mosquito feeding status and mosquito density on mosquito mortality. The outcome was the number of dead mosquitoes among all exposed mosquitoes (binary outcome). The known sources of variation were added as fixed effects: exposure of interest (temperature, feeding status or mosquito density), net type, cone replicate and day. Observation was included in the model as a random effect to account for clustering [31]. For the temperature assays that ran into the evening, time of day was also included as a fixed effect. Models were constructed with and without humidity, revealing that humidity did not affect the results or model fit; therefore, humidity was not included as a variable.

## Results

### Bioassay quality

In all experiments conducted, the mortality of the control was < 5% at 24, 48 and 72 h post-exposure. For the temperature experiments, the humidity was consistently maintained at around 65% [median: 65%, interquartile range (IQR): 58–68%). Similarly, during the feeding status experiments, the humidity level was sustained at approximately 82% (median: 82%, IQR: 74–83%). Lastly, for the density experiment, humidity was kept stable at about 80% (median: 80%, IQR: 77–82%). The holding temperature and RH humidity were 27 ºC ± 2 ºC and 60–100%, respectively, for all experiments (Additional file 1: Table S1).

### Effect of exposure temperature on *An. funestus* mortality

**Fig. 1:**
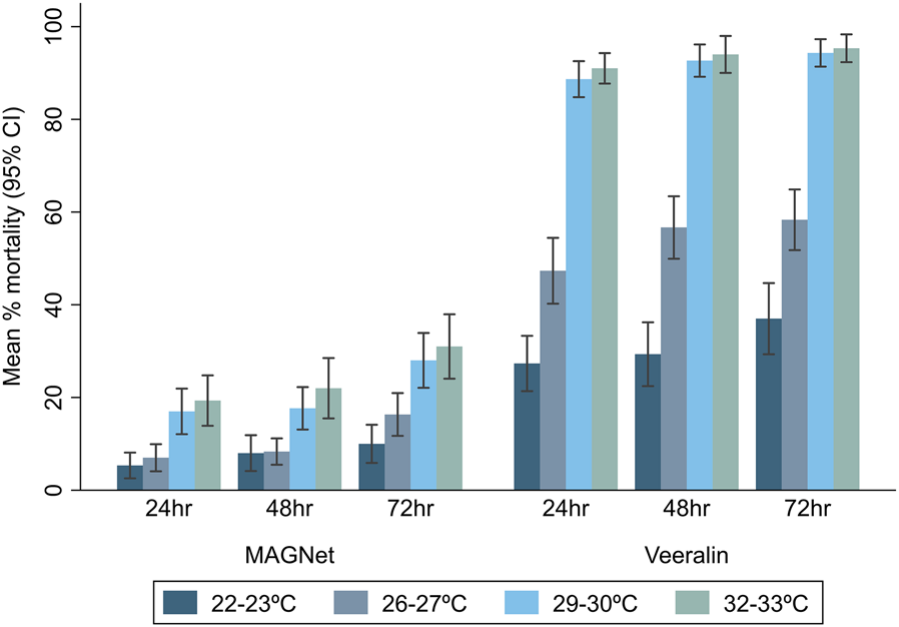
Mean percentage mortality of *Anopheles funestus* measured on cone bioassays at different temperature ranges

The results show that both net types exhibited temperature-dependent effects, with the mortality rates of *An. funestus* increasing with increasing temperature. The Veeralin ITN consistently showed higher mortality rates than the MAGNet ITN, especially at higher temperatures (Fig. 1). Compared to the mortality rate of *An. funestus* mosquitoes at the standard temperature regimen (26–27 °C), the proportion of deaths of mosquitoes was significantly higher at 29–30 °C and 32–33 °C for both nets (Table 1). At 22–23 °C, mosquito mortality was lower compared to that at the standard temperature [odds ratio (OR): 0.83, 95% CI 0.39–1.78, *P* = 0.636] with the MAGNet ITN and significantly lower (OR: 0.47, 95% CI 0.31–0.73, *p* = 0.001) with the Veeralin ITN at the 24 h holding time. A similar pattern was observed at holding times of 48 and 72 h (Table 1).

**Table 1:** Effect of exposure temperature on *Anopheles funestus* mortality measured on cone bioassays

Temperature (˚C)	*n*	24 h mortality		48 h mortality		72 h mortality	
		OR^a^ (95% CI)	*P *value	OR^a^ (95% CI)	*P* value	OR^a^ (95% CI)	*P* value
*MAGNet*							
26–27	60	Reference		Reference		Reference	
22–23	60	0.83 (0.39–1.78)	0.636	1.10 (0.50–2.42)	0.812	0.55 (0.27–1.09)	0.085
29–30	60	2.93 (1.59–5.39)	0.001	2.49 (1.32–4.67)	0.005	2.08 (1.23–3.52)	0.007
32–33	60	3.96 (1.99–7.87)	< 0.001	3.27 (1.44–7.45)	0.005	2.22 (1.10–4.49)	0.027
*Veeralin*							
26–27	60	Reference		Reference		Reference	
22–23	60	0.47 (0.31–0.73)	0.001	0.29 (0.17–0.51)	< 0.001	0.46 (0.27–0.80)	0.005
29–30	60	12.59 (7.51–21.10)	< 0.001	16.83 (8.34–33.97)	< 0.001	21.25 (10.18–44.38)	< 0.001
32–33	60	22.20 (11.45–43.05)	< 0.001	28.20 (12.10–65.72)	< 0.001	36.81 (15.35–88.23)	< 0.001

*CI* Confidence interval,* ITN* insecticide-treated net, *n* number of replicates,* OR* odds ratio

^a^The ORs were derived from mixed-effects logistic regression adjusted for net type, cone replicate, experimental time and day of the experiment, with observation was included as a random effect

### Effect of feeding status on *An. funestus* mortality

**Fig. 2:**
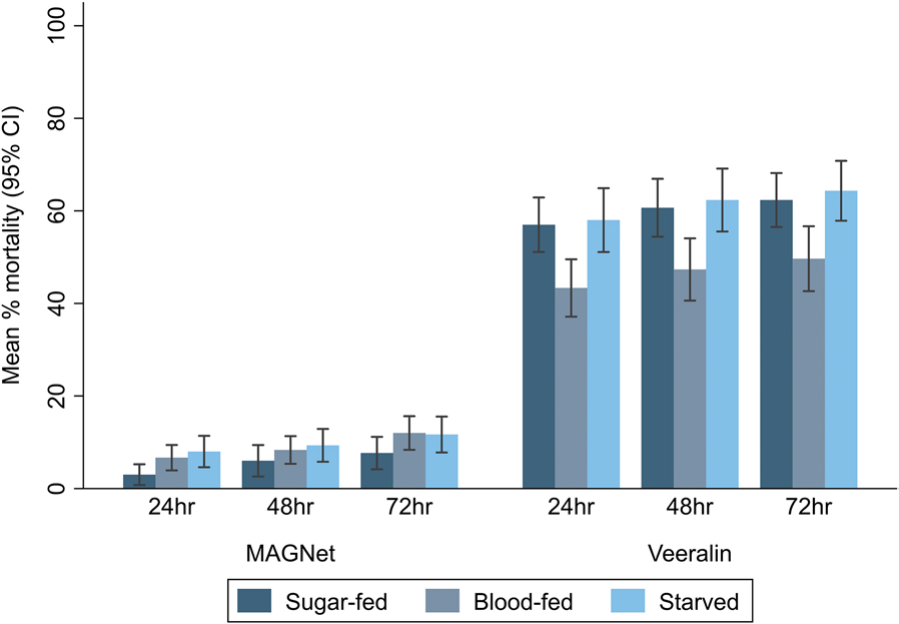
Mean percentage mortality of *An. funestus* measured on cone bioassays with different feeding statuses

The feeding status of mosquitoes had varying effects on the mortality of *An. funestus* depending with the ITN tested. The results of these experiments showed that there were higher mortality rates with the Veeralin ITNs than with the MAGNet ITNs at all feeding conditions (Fig. 2). For the MAGNet ITN, compared to sugar-fed mosquitoes at the 24 h holding time, blood-fed *An. funestus* showed similar mortality (OR: 2.23, 95% CI: 0.94–5.27, *P *= 0.068) while starved mosquitoes showed significantly higher mortality (OR: 2.88, 95% CI: 1.25–6.63, *P* = 0.013) (Table 2). Conversely, for the Veeralin ITN, compared to sugar-fed mosquitoes at 24 h, blood-fed mosquitoes showed a significantly lower mortality (OR: 0.56, 95% CI: 0.38–0.81, *P* = 0.002] and starved mosquitoes showed a similar mortality (OR: 1.05, 95% CI 0.72–1.51, *P* = 0.816). This mortality pattern was consistently observed at 48- and 72 h holding times (Table 2).

**Table 2:** Effect of mosquito feeding status on *An. funestus* mortality measured on cone bioassays

Feeding status	*n*	24 h mortality		48 h mortality		72 h mortality	
		OR^a^ (95% CI)	*P *value	OR^a^ (95% CI)	*P *value	OR^a^ (95% CI)	*P *value
*MAGNet *							
Sugar-fed	60	Reference		Reference		Reference	
Blood-fed	60	2.23 (0.94–5.27)	0.068	1.46 (0.75–2.85)	0.271	1.68 (0.95–2.97)	0.074
Starved	60	2.88 (1.25–6.63)	0.013	1.66 (0.86–3.21)	0.131	1.61 (0.91–2.86)	0.101
*Veeralin *							
Sugar-fed	60	Reference		Reference		Reference	
Blood-fed	60	0.56 (0.38–0.81)	0.002	0.55 (0.37–0.82)	0.003	0.58 (0.40–0.85)	0.005
Starved	60	1.05 (0.72–1.51)	0.816	1.08 (0.73–1.60)	0.703	1.10 (0.75–1.60)	0.637

*CI* Confidence interval,* ITN* insecticide-treated net, *n* number of replicates,* OR* odds ratio

^a^The ORs were derived from mixed-effects logistic regression adjusted for net type, cone replicate, experimental time and day of the experiment, with observation was included as a random effect

### Effect of mosquito density on *An. funestus* mortality

**Fig. 3:**
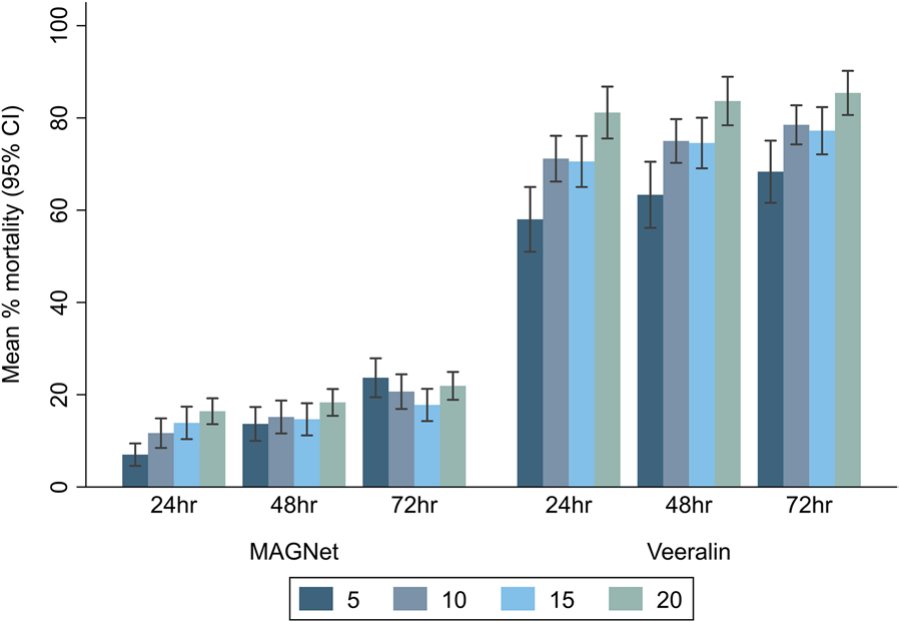
Mean percentage mortality of *An. funestus* measured on cone bioassays at different mosquito densities

Mortality rates consistently increased with increasing mosquito density from five mosquitoes per cone to 20 mosquitoes per cone, indicating a direct relationship between number of mosquitoes exposed in a cone and the observed mortality (Fig. 3). Compared to a density of five mosquitoes per cone, the odds of mortality significantly increased with 10 mosquitoes per cone (OR: 1.81, 95% CI: 1.03–3.20, *P* = 0.040), 15 mosquitoes per cone (OR: 2.16, 95% CI: 1.25–3.71, *P* = 0.006) and 20 mosquitoes per cone (OR: 2.74, 95% CI: 1.61–4.67, *P* < 0.0001), respectively, at 24 h post-exposure with the MAGNet ITN, although the differences were less pronounced at longer holding times. A similar trend was also observed with the Veeralin ITN at 24, 48 and 72 h post-exposure, with increased density resulting in a higher odds of mosquito mortality, with this trend remaining significant at longer holding times (Table 3).

**Table 3:** Effect of mosquito density on *An. funestus* mortality measured on cone bioassays

Mosquito density	*n*	24 h mortality		48 h mortality		72 h mortality	
		OR^a^ (95% CI)	*P *value	OR^a^ (95% CI)	*P *value	OR^a^ (95% CI)	*P *value
*MAGNet *							
5	60	Reference		Reference		Reference	
10	60	1.81 (1.03–3.20)	0.040	1.14 (0.72–1.80)	0.578	0.83 (0.57–1.21)	0.334
15	60	2.16 (1.25–3.71)	0.006	1.07 (0.69–1.65)	0.778	0.68 (0.48–0.98)	0.037
20	60	2.74 (1.61–4.67)	< 0.001	1.44 (0.94–2.21)	0.090	0.90 (0.64–1.26)	0.539
*Veeralin *							
5	60	Reference		Reference		Reference	
10	60	2.06 (1.24–3.42)	0.005	2.03 (1.16–3.57)	0.013	1.94 (1.11–3.39)	0.019
15	60	2.08 (1.27–3.42)	0.004	2.15 (1.23–3.73)	0.007	1.91 (1.11–3.28)	0.020
20	60	5.05 (3.03–8.43)	< 0.001	4.89 (2.78–8.61)	< 0.001	4.10 (2.35–7.15)	< 0.001

*CI* Confidence interval,* ITN* insecticide-treated net, *n* number of replicates,* OR* odds ratio

^a^The ORs were derived from mixed-effects logistic regression adjusted for net type, cone replicate, experimental time and day of the experiment, with observation was included as a random effect

## Discussion

The WHO cone bioassay serves as a cornerstone for the evaluation of insecticide-based vector control tools. Despite its standardized nature, there is a critical need to comprehensively investigate how changes to different parameters can impact the outcome of the tests. This study was conducted to investigate how alterations to parameters such as temperature, feeding status and mosquito density would affect the outcome of the bioassay.

Although the standard cone bioassay is conventionally conducted at a controlled temperature of 27 °C ± 2 °C, we deliberately explored temperature ranges both below and above this standard in our experiments. Our findings revealed a notable influence of temperature on *An. funestus* mortality rates for both pyrethroid and pyrethroid-PBO ITNs. High temperatures were associated with increased mosquito mortality, while lower temperatures led to decreased mortality. A similar trend was observed in tube assays where the effects of exposure to permethrin-impregnated nets were found to be greater at higher temperatures among susceptible and pyrethroid-resistant malaria vectors [18]. This observation suggests the presence of a positive temperature coefficient, indicating that an insecticide becomes more toxic with increasing temperature [32].

Additionally, the intensity of the impact of temperature on mosquito mortality appeared to vary across the different ITN classes examined. Notably, the Veeralin ITN consistently exhibited more pronounced and, consequently, statistically significant effects at higher temperatures compared to the MAGNet ITN. This difference may be attributed to the inclusion of PBO in the Veeralin ITN. PBO is a synergist known to inhibit metabolic enzymes responsible for detoxifying insecticides; this action, in turn, restores the insecticidal potency of ITNs against metabolically pyrethroid-resistant malaria vectors [33]. In a related study by Glunt et al. [15], the interaction between PBO and temperature was investigated using the standard WHO tube test; however, the findings of this study were unable to determine whether temperature influenced the restoration of pyrethroid susceptibility in resistant malaria vectors exposed to PBO because all mosquitoes exposed to PBO and subsequently to deltamethrin died [15]. One possible explanation for the increased toxicity of PBO at higher temperatures in our study may be an increased reaction rate of irreversible inhibition under these conditions [15]. An alternative hypothesis is that insecticides are more bioavailable at higher temperatures, which may be the case for PBO as it is a liquid (Ole Skovmand, personal communication).

Data from this experiment adds to the body of evidence showing that temperature must be carefully considered when testing insecticides against biological systems. A bimodal temperature-activity distribution has been reported for several insecticides and mosquito species [34–37], and 27 ºC ± 2 ºC provides a conservative measurement of mortality. Temperature affects the way in which pyrethroids work in insects. The toxicity of pyrethroids [32] and of other insecticide classes [38] is positively correlated with temperature. Similarly, the sensitivity of the mosquito nervous system and mosquito immune responses and metabolic activities are all affected by temperature, thereby influencing the efficacy of insecticides [39, 40]. Generally, neuron sensitivity declines between temperatures of 30 ºC and 35 ºC, which influences the efficacy of insecticides [41]. Elevated temperatures can potentially shorten mosquito lifespan by accelerating the reaction rates of diverse metabolic processes that influence both development and life history [41]. Furthermore, the permeability of the insect cuticle, a crucial barrier to insecticide penetration, may be influenced by temperature. Higher temperatures might enhance cuticular permeability, potentially leading to a more rapid and efficient uptake of insecticides [42]; hence, higher mortality is associated with increasing temperature. The availability of active ingredients to the surface of the net is accelerated by heat; consequently, with an increase in temperature, the active ingredients reach the surface of the ITN more rapidly, enhancing their bioavailability; this, in turn, leads to a heightened uptake of insecticide by mosquitoes [43].

There is some evidence that humidity can also affect mosquito mortality after insecticide exposure [44], and humidity is known to affect mosquito survival [45]; consequently, humidity should be carefully maintained during mosquito holding post-exposure. In the present study, humidity was held constant throughout the experiments (Additional file 1: Table S1), although further studies may be warranted to explore the effect of humidity variations on the mortality rate observed in bioassays.

Feeding status of mosquitoes is yet another dynamic factor that can affect their interaction with insecticides. In the standard bioassay [8], mosquitoes are sugar-fed before exposure to insecticides. However, in the present study, we evaluated the results of bioassays conducted with starved and blood-fed mosquitoes for the interaction with ITNs. The results revealed that the feeding status of mosquitoes impacts the outcome of cone bioassays, with the variation found for both the pyrethroid-only and the pyrethroid-PBO ITNs. We observed that with the pyrethroid ITN, sugar-fed mosquitoes had a mortality rate similar to that of blood-fed mosquitoes and a lower mortality rate than that of starved mosquitoes, although the confidence intervals of the ORs were wide in all estimates, presumably because of low observed mortality in the pyrethroid ITN arm of the study. Norris et al. [21] investigated the relationship between mosquito feeding status and susceptibility status in tube tests; similar to our findings, these authors reported that the percentage mortality after permethrin exposure was significantly higher in starved mosquitoes than in sugar-fed mosquitoes.

Interestingly, in the present study, blood-fed mosquitoes had lower mortality rates than sugar-fed mosquitoes for the pyrethroid-PBO net. It has been reported that blood meals stimulate insecticide detoxification mechanisms in pyrethroid-resistant *An. funestus* mosquitoes, leading to increased insecticide tolerance in bottle bioassays [16]. Furthermore, a single blood meal has been found to significantly reduce insecticide-induced mortality to pyrethroid and dichlorodiphenyltrichloroethane (DDT) relative to sugar-fed resistant *An. arabiensis* in the WHO susceptibility test [46]. Similarly, in recent studies it has been observed that blood-feeding increases insecticide tolerance among blood-fed susceptible *Anopheles gambiae* [47] and enhances the levels of resistance to deltamethrin among resistant blood-fed *An. funestus* [16]. The lower mortality rates observed with blood-fed mosquitoes in the present study with the pyrethroid-PBO ITN may suggest that the ingestion of the blood meal triggers oxidative stress and boosts metabolic acitivity in mosquitoes [48, 49], potentially resulting in increased expression of detoxification enzymes [50] and affecting the toxic dose mosquitoes receive from insecticide exposure [51]. However, additional research is needed to investigate the relationship between pyrethroid-PBO ITNs and blood meals.

A study by Aizoun [52] demonstrated that 2- to 5-day-old blood-fed *An. gambiae* mosquitoes exhibited lower mortality rates in comparison to 20-day-old unfed mosquitoes. Further investigations revealed that, preceding exposure to deltamethrin, younger *An. gambiae* mosquitoes (2–5 days old) displayed reduced mortality rates compared to their older counterparts (14–16 days old), irrespective of their feeding status [47]. This finding underscores the role of age as a contributing factor influencing both insecticide toxicity and mosquito mortality. Consistent with these observations, based on the results of a study published in 2012, Chouaibou et al. [53] reported that mortality rates were notably lower among 2- and 3-day-old mosquitoes than in their 10-day-old counterparts following exposure to insecticide. These collective findings emphasize the significance of mosquito age in shaping responses to insecticide exposure, thereby influencing overall mortality outcomes.

The present study also showed that higher mosquito density was associated with increased mortality. This effect of density may be likely due to increased competition for available space within the cone; as mosquitoes crowd together, they interact more and are more likely to encounter and pick up insecticides from the treated surfaces. The effects of cage size and density have also been shown to influence the duration of insect repellent efficacy [54], presumably due to the probability of mosquitoes encountering a host. Increased activity in cone tests is associated with increased mortality in video cone tests, even in the control [55], and may be influenced by mosquito interactions with others in the confined cone.

It should be noted that our study focused exclusively on assessing the impact of temperature, mosquito feeding status and mosquito density within the scope of cone bioassays; consequently, any generalizability of these findings to broader contexts, such as susceptibility tests, may be limited. Additionally, the bioassays were conducted using only one mosquito strain, namely pyrethroid-resistant *An. funestus* mosquitoes, and only alpha-cypermethrin ITNs. To enhance the comprehensiveness of the results, future investigations needed to include a broader range of both susceptible and resistant mosquito vectors, as well as a greater diversity of active ingredients. Similar studies are essential for gaining a more comprehensive perspective on the interplay between parameters that could influence insecticide susceptibility assessments and other types of bioassays routinely deployed for vector control product assessment, such as tunnel tests.

## Conclusions

The study highlights that the testing parameters of exposure temperature, mosquito feeding status and mosquito density significantly influence the outcome of cone bioassays. Based on our results, it is evident that even a slight temperature variation significantly influenced mosquito mortality; consequently, when reporting the results of cone bioassays, the precise testing conditions used must be given, especially if they deviate from the recommended temperature of 27 °C ± 2 °C. In addition, deviation from the recommended five mosquitoes per cone also strongly impacted the results of the cone tests reported in the present study. Careful adherence to testing parameters outlined in WHO ITN testing guidelines will likely improve the repeatability of studies within and between product testing facilities. Additionally, while maintaining the use of sugar-fed mosquitoes in cone bioassays is necessary for standardization, further investigations are warranted to understand the interactions between blood meals and insecticide detoxification as these may impact on the efficacy of vector control tools.

### Supplementary Information

**Additional file 1: **Median (IQR) temperature and humidity throughout the experiment and the holding period.

**Additional file 2: **Cone bioassay data on the effect of exposure temperature, feeding status and mosquito density on *An.funestus* mortality.

## Data Availability

The data supporting these findings are available in Additional file 2.

## Abbreviations

AI: Active ingredients
IHI: Ifakara Health Institute
IRS: Indoor residual spraying
ITNs: Insecticide-treated nets
PBO: Piperonyl butoxide
VCPTU: Vector Control Product Testing Unit
