# Supplementary material for: Temperature, mosquito feeding status and mosquito density influence the measured bio-efficacy of insecticide-treated nets in cone assays
Source: Parasit Vectors. 2024 Mar 28;17:159. doi: 10.1186/s13071-024-06210-y (PMC10979578; doi:10.1186/s13071-024-06210-y)
Supplement: Supplementary file 1 — Additional file 1: Median (IQR) temperature and humidity throughout the experiment and the holding period. [file 13071_2024_6210_MOESM1_ESM.docx]

|  | Exposure time | | | | Holding time | | | |
| --- | --- | --- | --- | --- | --- | --- | --- | --- |
|  | Temperature (˚C) | | Relative Humidity | | Temperature (˚C) | | Relative Humidity | |
|  | Median | Interquartile range (IQR) | Median | Interquartile range (IQR) | Median | Interquartile range (IQR) | Median | Interquartile range (IQR) |
| Effect of exposure temperature on *Anopheles funestus* mortality experiment | 28.5 | 25.2, 31.3 | 65.3 | 58.2, 68.1 | 25.8 | 25.6, 26.0 | 79.0 | 73.7, 84.6 |
| Effect of feeding status on *Anopheles funestus* mortality experiment | 26.0 | 25.7, 27.1 | 81.7 | 74.2, 82.9 | 26.3 | 25.9, 26.8 | 80.0 | 77.6, 82.3 |
| Effect of mosquito density on *Anopheles funestus* mortality experiment | 26.8 | 26.7, 27.4 | 79.8 | 76.5, 82.1 | 26.6 | 26.1, 26.9 | 78.4 | 74.5, 80.6 |

Table S1: Median (IQR) temperature and humidity throughout the experiment and the holding period.
